# Artificial Visual Information Produced by Retinal Prostheses

**DOI:** 10.3389/fncel.2022.911754

**Published:** 2022-06-06

**Authors:** Sein Kim, Hyeonhee Roh, Maesoon Im

**Affiliations:** ^1^Brain Science Institute, Korea Institute of Science and Technology, Seoul, South Korea; ^2^School of Electrical Engineering, College of Engineering, Korea University, Seoul, South Korea; ^3^Division of Bio-Medical Science & Technology, KIST School, University of Science and Technology, Seoul, South Korea

**Keywords:** retinal prosthetics, visual information, neural computation, information theory, spike trains

## Abstract

Numerous retinal prosthetic systems have demonstrated somewhat useful vision can be restored to individuals who had lost their sight due to outer retinal degenerative diseases. Earlier prosthetic studies have mostly focused on the confinement of electrical stimulation for improved spatial resolution and/or the biased stimulation of specific retinal ganglion cell (RGC) types for selective activation of retinal ON/OFF pathway for enhanced visual percepts. To better replicate normal vision, it would be also crucial to consider information transmission by spiking activities arising in the RGC population since an incredible amount of visual information is transferred from the eye to the brain. In previous studies, however, it has not been well explored how much artificial visual information is created in response to electrical stimuli delivered by microelectrodes. In the present work, we discuss the importance of the neural information for high-quality artificial vision. First, we summarize the previous literatures which have computed information transmission rates from spiking activities of RGCs in response to visual stimuli. Second, we exemplify a couple of studies which computed the neural information from electrically evoked responses. Third, we briefly introduce how information rates can be computed in the representative two ways – direct method and reconstruction method. Fourth, we introduce *in silico* approaches modeling artificial retinal neural networks to explore the relationship between amount of information and the spiking patterns. Lastly, we conclude our review with clinical implications to emphasize the necessity of considering visual information transmission for further improvement of retinal prosthetics.

## Introduction

Vision is unarguably the most critical sensory modality (Hutmacher, 2019) among the five senses of humans. Diverse causes can result in low vision or blindness, and there have been numerous attempts to restore sight to blind individuals by electrically stimulating visual cortex (Dobelle et al., 1974, 1976), lateral geniculate nucleus (Pezaris and Reid, 2007), optic nerve (Gaillet et al., 2019), or retina (Humayun et al., 1996). In the case of retina, the electric stimulation is effective to elicit artificial visual percepts for outer retinal degenerative diseases such as retinitis pigmentosa and age-related macular degeneration, which primarily damage photoreceptors (Bunker et al., 1984; Curcio et al., 1996). Microelectronic retinal prostheses including commercialized ones (e.g., Argus II, Alpha-IMS/AMS, and PRIMA) reported some promising clinical outcomes by electrically stimulating the remaining inner retinal neurons (Humayun et al., 1996; Rizzo et al., 2003; Fujikado et al., 2007; Zrenner et al., 2011; da Cruz et al., 2013; Dorn et al., 2013; Stingl et al., 2013; Shivdasani et al., 2017; Palanker et al., 2020). However, the best visual acuity of elicited artificial vision (20/460) (Palanker et al., 2020) is still far below the level of legal blindness (20/200) as well as normal vision (20/20). To achieve high-resolution visual prosthetics, various research groups have tried to electrically activate cells in only targeted small areas using hardware and software approaches. For example, several groups proposed novel designs of microelectrodes (Flores et al., 2018, 2019; Seo et al., 2020) or photovoltaic arrays (Ferlauto et al., 2018; Wang et al., 2021) to further localize electric current in a smaller region. Also, Jepson et al. (2014) used spatially patterned electric stimulation, and Weitz et al. (2015) demonstrated retinal activation in much smaller area with 25 ms-long pulses.

The other important research topic in retinal prosthetics has long been the cell-type specific stimulation. As the starting point of the most complex sensory system, and the mammalian retinas have numerous types of retinal ganglion cells (RGCs) which are the output spiking neurons sending neural signals to the downstream visual centers (Masland, 2001; Rockhill et al., 2004; Sanes and Masland, 2015; Baden et al., 2016). Among those types, ON and OFF RGCs are known to play a critical role in forming visual percepts (Schiller et al., 1986; Schiller, 1992). In addition to the asymmetricities between light-evoked responses of the ON vs. the OFF pathways (Ölveczky et al., 2003; Margolis and Detwiler, 2007; Liang and Freed, 2012; Freed, 2017), retinal prosthetic studies reported contrasting differences between the two pathways (Freeman et al., 2010; Kameneva et al., 2010; Twyford et al., 2014; Im and Fried, 2015, 2016a; Lee and Im, 2019). However, given the unique mosaic arrangement of each type of RGCs (DeVries and Baylor, 1997; Masland, 2012), it seems almost inevitable to activate every type of RGCs located near a given electrode delivering electric stimulation. Nevertheless, it seems reasonable to aim more biased activation of the ON system because the earlier clinical trials reported dominantly bright sensation (Humayun et al., 1996, 2003; Fujikado et al., 2007; Naycheva et al., 2012). Recent studies demonstrated the ON/OFF response ratio can be increased by modulating several stimulus parameters such as stimulus durations (Im et al., 2018), repetition rates (Cai et al., 2011, 2013; Twyford et al., 2014; Im and Fried, 2016a), waveform shapes (Lee and Im, 2018), and current amplitudes (Lee and Im, 2019). The use of penetrating microelectrode to the specific stratification depth may enhance the cell-type specific activation of either ON or OFF RGCs (Roh et al., 2022). To date, however, it appears extremely challenging to achieve completely selective stimulation of targeted cell type(s) using electrical stimulation. In contrast, optogenetic approaches can selectively activate the ON pathway (Lagali et al., 2008; Gaub et al., 2014; Macé et al., 2015; Lu et al., 2016) but they need to address potential phototoxicity (Grimm et al., 2001; van Wyk et al., 2015; Simunovic et al., 2019) and/or low transfection efficiency of viral vectors (Lagali et al., 2008; Busskamp and Roska, 2011).

**FIGURE 1:**
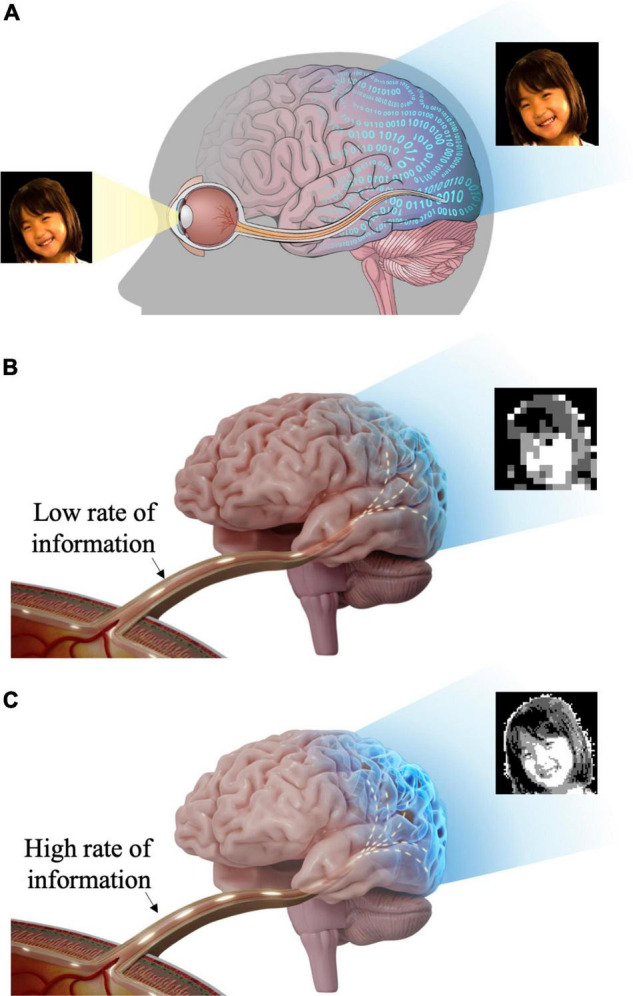
Schematic illustration of visual information transfer from the eye to the visual cortex. The visual information flows through the lateral geniculate nucleus (LGN) *en route* to the visual cortex. But, LGN is not shown in this figure for brevity. **(A)** Retinal ganglion cells generate spiking activities to visual stimuli and transmit visual information to the brain (visual cortex) through the optic nerve and optic radiation. **(B)** Insufficient visual information and less natural artificial vision may activate higher visual centers inappropriately. **(C)** Rich visual information and more natural artificial vision may activate higher visual centers more effectively.

In addition to the aforementioned two important features (i.e., the spatial resolution and the cell type-specific stimulation), it may be critical to assess whether retinal prostheses restore ample enough information. It is because the remarkably complex retinal circuits compress the visual world in real-time (Kolb, 2003), making RGCs transmit an incredible amount of visual information to the brain (Figure 1A), which could be estimated as big as 875,000 bits/s (52.5 Mb/min) (Koch et al., 2006). Thus, less artificial information may confound the brain, resulting in unclear artificial visual percepts (Figure 1B), whereas more transferred information may help the brain more precisely recognize artificial visual images (Figure 1C). However, there has been a limited number of retinal prosthetic researches which had studied neural information aspects of electrically evoked spiking activities (Eger et al., 2005; Kang et al., 2021). Contrastingly, in the case of cochlear implants which show great clinical success, information transmission in the auditory system has been well studied (Mino, 2007; Zeng et al., 2008; Hannan et al., 2012; Moroz et al., 2012; Gao et al., 2013). Analyzing the RGC spiking activities using information theory is likely to help understanding how the brain deciphers artificial visual information (Quian Quiroga and Panzeri, 2009). In particular, it has been known that retinal spike trains are precisely structured to efficiently convey visual information (Berry et al., 1997). Therefore, other than simply re-activating RGCs to send spike trains to downstream neurons, it would be essential to understand how much information is encoded (Timme and Lapish, 2018) by the ensemble of prosthetically evoked spikes arising in RGCs (Freeman et al., 2011) to estimate the quality of artificial vision. Also, information researches are likely to offer valuable insights for the improved performance of retinal implants.

## Neural Information of Intrinsic Visual Responses in the Healthy Retina

The retina divides the complex visual world into several parallel pathways (Wässle, 2004; Roska et al., 2006; Nassi and Callaway, 2009) using the remarkable variety of RGC types (Baden et al., 2016). All RGCs in mammalian retinas encode visual information by spatiotemporal structure of spike trains but differently across the RGC types (Berry et al., 1997; Masland, 2001; Field and Chichilnisky, 2007; Zeck and Masland, 2007; Sanes and Masland, 2015). Much work has studied the relationship between spiking and information rates in RGC responses to light stimuli using several computational methods (Koch et al., 2004, 2006; Passaglia and Troy, 2004; Kang et al., 2021) (see a later section regarding how direct and reconstruction methods compute neural information). Particularly, it is notable that distinct RGC types showed different information rates (Koch et al., 2006). For example, in guinea pig retinas, two groups of RGCs with brisk or sluggish responses transmitted information of 21 ± 9 and 13 ± 7 bits/s (*n* = 19 and *n* = 23 cells), respectively (Koch et al., 2004). More recently, Kang et al. (2021) analyzed the amount of neural information conveyed through brisk transient (BT) and brisk sustained (BS) subtypes of ON and OFF pathways in rabbit retinas. In responses to spot flashes, 1.83 ± 0.07 and 1.89 ± 0.04 bits/s of information were transmitted by ON BT and OFF BT cells, respectively; while 2.53 ± 0.08 and 3.00 ± 0.22 bits/s of information were transmitted by ON BS and OFF BS cells (*n* = 15 for each type), respectively.

Compared to laboratory stimuli such as stationary spot flashes, natural stimuli are known to evoke sparser spiking activities in RGCs (Kayser et al., 2003; David et al., 2004; Felsen et al., 2005; Puchalla et al., 2005; Touryan et al., 2005; Talebi and Baker, 2012; Im and Fried, 2016b). For example, about 65% of RGCs do not fire constantly in response to natural stimuli, and it has been argued that the sparse coding can reduce the number of activated neurons, thereby saving energy for information transmission (Wang et al., 2018). Given that natural stimuli contain much bigger visual information than laboratory stimuli, it seems important to compare both amount and efficiency of information conveyed in responses arising from laboratory vs. natural stimuli.

## Artificial Visual Information of Electrically Evoked Responses in the Degenerate Retina

Aforementioned examples suggest that, for high-quality artificial vision, it may be crucial to study whether electrically elicited spiking activities of RGCs in the degenerate retina convey visual information at a similar level of visually evoked responses arising in the normal retina (Fried et al., 2006; Freeman et al., 2011). Surprisingly, however, there are very few studies that have discussed the neural information produced by electric stimulation. For example, Eger et al. (2005) stimulated the cat retina electrically while recording neuronal activities at 15 sites in the visual cortex. They estimated 20–160 bits/s of information was transferred at a single recording site when a single electrode was activated (Eger et al., 2005). They also reported 500 bits/s of information was transmitted at 15 recording sites when seven electrodes were activated. However, the information rates varied noticeably between experiments depending on the positions of stimulation and recording sites since it was difficult to place electrodes accurately at corresponding retinotopic loci.

Another example investigated the amount of information elicited by both light and electric stimulation in more sophisticated ways (Kang et al., 2021). First, they classified RGCs into the four major types (i.e., BT and BS subtypes of ON and OFF pathways) in the healthy rabbit retina, and then analyzed spiking activities in each type to compare the neural information between light and electric responses of identical sets of RGCs. When the number of cells increased up to 15, electric responses of ON BT and BS RGCs displayed a similar level of neural information with their light responses, whereas electrically evoked responses of OFF BT and BS cells showed greatly reduced information than those of their light responses. Second, they tried to correlate the neural information and the cell-to-cell heterogeneity of spiking responses. Interestingly, the ON RGCs showed similarly heterogeneous responses regardless of light and electric stimuli. In contrast, the OFF RGCs showed much more homogeneous responses to electric than light stimuli. The reduced information by the homogeneous responses of OFF RGCs is consistent with previous studies which reported the amount of neural information increases with a higher cell-to-cell heterogeneity in naturally evoked neural activities (Chelaru and Dragoi, 2008; Padmanabhan and Urban, 2010). However, it is also noteworthy that there is an optimal level of population response heterogeneity which maximizes the transmission of neural information as well as minimizes the effect of external noises (Tkačik et al., 2010; Tripathy et al., 2013; Im and Kim, 2020). Kang et al. (2021) also demonstrated the ultimate heterogeneity (i.e., completely random spiking) of RGC responses is not ideal to transfer neural information because they are less immune to noise: they showed the increased trial-to-trial variability (i.e., jitter) reduces the population neural information more substantially in random spiking responses than in physiological RGC responses which showed intermediate levels of the cell-to-cell heterogeneity. Given an earlier study that reported RGC spiking consistency is gradually reduced with advancing retinal degeneration (Yoon et al., 2020), the analyses of Kang et al. (2021) suggest that the amount of information transmitted to the brain decreases as the retina degenerates. For further improvement of retinal prosthetics, it may be critical to investigate how information rates can be enhanced in degenerate retinas by altering electric stimulation conditions.

## Two Methods for Neural Information Calculation

**FIGURE 2:**
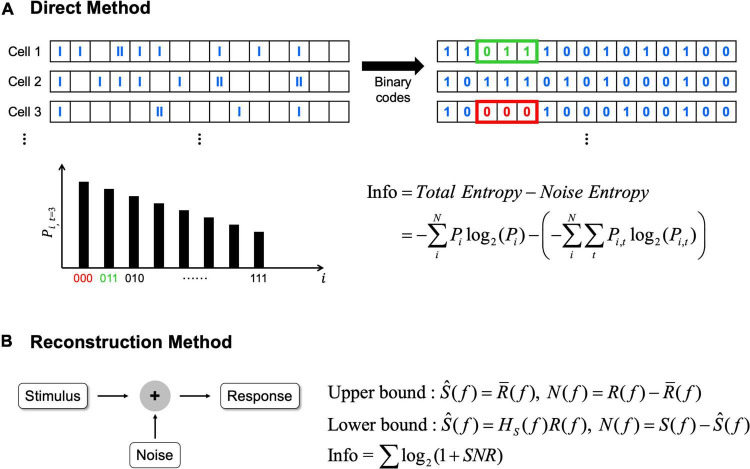
The direct method and the reconstruction method can be applied to calculate information rates. **(A)** In the direct method, average information rates are the difference between total entropy and noise entropy. *N* represents the total number of possible binary code combinations, *i* represents binary code combination. *P*_*i*_ indicates probability of particular binary code combinations and similarly *P*_*i,t*_ indicates the probability of particular binary code combinations at a specific time, *t*. **(B)** In the reconstruction method, average information rates are obtained from the signal to noise ratio (SNR). Signal and noise are calculated differently in each bound (see Passaglia and Troy, 2004 for how signal and noise are calculated). In here, *S(f)* means signals which are the Fourier transforms of the stimulus, *R(f)* means responses which are also the Fourier transforms of the response, respectively. *Ŝ(f)* means the best estimate of stimulus. In upper bound, *Ŝ(f)* is obtained by averaging *R(f)* [i.e,. *R̄(f)*]. In lower bound, *Ŝ(f)* is obtained by the linear decoder filter, *H_*s*_(f)*. *N(f)* represents noise, and noise is also different in each bound. In upper bound, noise is the difference between response and average response, while noise is the difference between signals and estimated stimulus in lower bound.

Information theory can quantify how much information about a given external stimulus is conveyed by neural responses; and there are two representative ways to compute the information from neural spike trains (Borst and Theunissen, 1999; Passaglia and Troy, 2004). First, direct method can be applied to calculate average information transmitted by the difference between total entropy of the neural response and noise entropy (Figure 2A; Koch et al., 2004; Osborne et al., 2008; Stone, 2018; Kang et al., 2021). In this method, before calculating the entropy, spikes of each cell are allocated into time bins in a fixed duration which may differ depending on experimental methods (Koch et al., 2004). Then, if one or more spikes are present in a given time bin, 1 is assigned; while 0 is assigned if there are no spikes (Osborne et al., 2008; Kang et al., 2021). Before using this binary code array to calculate entropy, it is important to choose an appropriate length of binary code combinations depending on the particular context being experimented (Theunissen and Miller, 1995). Then, total entropy is calculated from the probability of particular binary code combinations in the entire recording. Similarly, noise entropy is also estimated but from the probability of particular binary code combinations at a specific given time relative to the identical stimuli (*see* Osborne et al., 2008; Stone, 2018 for more details).

Second, reconstruction method can be used which is a variant of the abovementioned direct method (Figure 2B; Borst and Theunissen, 1999). The reconstruction method can determine the neural responses based on the stimulus or predict what stimulus is given based on the response (Eger and Eckhorn, 2002; Victor, 2006). Unlike the direct method requiring no assumption, the reconstruction method estimates upper or lower bound of information depending on assumptions. When we assume neural responses have a Gaussian distribution over the frequency range then the upper bound is placed since Gaussian distribution has the maximum entropy (Borst and Theunissen, 1999; Passaglia and Troy, 2004). Alternatively, the lower bound is placed when we assume information can be decoded linearly to estimate the best possible stimulus from the neural responses (Borst and Theunissen, 1999). Because we cannot include all of the information with this assumption since neural responses are predominantly non-linear and Poisson process (Felsen and Dan, 2005), it becomes the lower bound of the information. For the computation of information rates using the reconstruction method, the signal to noise ratio in the frequency domain must be calculated (see Passaglia and Troy, 2004 for more details).

In comparison between these two methods, the direct method needs a lot of experimental data to calculate since it has no assumptions with signals and it does not reveal which stimulus aspects are being encoded. In sharp contrast, the reconstruction method needs significantly less data than the direct method, making it useful for the field that has limited amount of available data.

## Recent *In Silico* Computational Neuroscience Approaches for Neural Information Analysis

As an alternative to population responses recorded *in vivo* or *in vitro*, we can simulate population codes using computational tools. A few preceding studies suggest that the artificial retina model helps understand how the retina responds to stimuli and how the retina encodes visual information (Wohrer et al., 2006; Pei and Qiao, 2010). Also, Brette (2009) introduced an *in silico* approach to generate population codes based on the designed firing rate and pairwise correlation of spike trains. With the computational approaches using artificial spike trains, it is possible to more precisely analyze the relationship between the spiking elements and neural information.

Recently, Roh et al. (2021) used a modified version of “Brian 2” (Brette, 2009) to generate various sets of spike trains which have different levels of correlations; spike time tiling coefficients (STTCs) were calculated to quantify the correlations across spike trains (Cutts and Eglen, 2014). Then, they analyzed neural information as a function of the correlation level for a wide range of average STTC values. According to the abovementioned study of Roh et al., the increased spiking heterogeneity across cells can enhance information transmission. Earlier, Hunsberger et al. (2014) reported the heterogeneity may better encode the stimulation by expressing complementary aspects of stimuli. In addition to the cell-to-cell spiking heterogeneity, other spiking features may be also crucial in better encoding visual information. For example, as a follow-up study of Roh et al. (2021) and Kim et al. (2022) further explored the relationship between information and other spiking elements such as mean firing rate and spiking duration. These *in silico* approaches may expedite future studies regarding how different stimulation parameters of retinal prostheses make RGCs transmit sufficient information to the brain.

## Clinical Implications of Artificially Evoked Visual Neural Information

Other than the aforementioned studies (Eger et al., 2005; Kang et al., 2021; Roh et al., 2021; Kim et al., 2022), retinal prosthetics has not paid enough attention on how their microelectronic devices and/or stimulation strategies improve electrically produced neural information. Although there is no direct clinical evidence supporting that prosthetic responses which transmit more information would be better perceived, it is important to note a recent sight restoration study demonstrated better animal behavior responses when the restored spiking activities were more heterogeneous across RGCs (Berry et al., 2017). Given the correlation between the cell-to-cell spiking heterogeneity and the transmitted neural information (Tripathy et al., 2013; Kang et al., 2021), the improved behavior of the animals is likely due to the enhanced visual information transmission. The optical stimulation using optogenetic approaches (Bi et al., 2006; Sahel et al., 2021), photoswitch compound (Tochitsky et al., 2016), and photoactivatable G protein-coupled receptor (Berry et al., 2017) must be promising vision restoration methods because they would not require any surgical implantation of microelectronic devices. However, in those fields as well, it is difficult to find analyses of artificially evoked neural information.

In the past clinical trials of microelectronic retinal prostheses, the most prosthetic users with retinitis pigmentosa were at the near-end stage of degeneration, who showed no light perception or hand motion (Humayun et al., 2003; Zrenner et al., 2011; Stingl et al., 2013; but, see Palanker et al., 2020, 2022 for prosthetic users with age-related macular degeneration who had still periphery vision). In such an advanced stage, their retinas were likely to send less neural information to the brain in response to electric stimulation. It is because both direct and indirect activation of RGCs are likely to result in low information transmission rates as follow: (1) direct activation which can precisely elicit spike at intended timing may produce too homogeneous spiking across RGCs compared to natural spiking activity, and (2) indirect activation generates highly inconsistent (i.e., big trial-to-trial variability in each RGCs) network-mediated responses in severely degenerate retinas (Yoon et al., 2020), increasing noise which reduces information transmission (Kang et al., 2021). Numerous previous literatures have studied synchronous/correlated spiking activities of neighboring RGCs in responses to visual stimuli (Meister et al., 1995; DeVries, 1999; Puchalla et al., 2005; Shlens et al., 2008). Depending on the extent of response synchrony/correlation of neighboring RGCs, the brain might recognize different visual messages as argued earlier (Puchalla et al., 2005). However, systematic understanding is still lacking regarding electrically evoked responses, raising the need for such measurements.

## Conclusion

The retina is remarkably complicated in both anatomic and functional aspects. Given the incredible complexity of the retina, it seems insufficient to simply make RGCs fire again for high-quality artificial vision. However, the field of retinal prosthetics has paid little attention on how much artificial visual information could be created by implanted retinal prostheses and transmitted from the retina to the brain, compared to other aspects of electrically evoked retinal responses such as spike counts, firing rates, and so on. For the further improved quality of prosthetic vision, it may be critical to explore whether sufficient amount of visual information is transmitted from the retina to the brain. Probably, more information enhances the perception quality of artificial vision.

## Author Contributions

SK and HR contributed to drafting the manuscript and figures design. MI conceived the work and revised the manuscript. All authors read and approved the final manuscript.

## Conflict of Interest

The authors declare that the research was conducted in the absence of any commercial or financial relationships that could be construed as a potential conflict of interest.

## Publisher’s Note

All claims expressed in this article are solely those of the authors and do not necessarily represent those of their affiliated organizations, or those of the publisher, the editors and the reviewers. Any product that may be evaluated in this article, or claim that may be made by its manufacturer, is not guaranteed or endorsed by the publisher.
